# Network analysis of the association between social anxiety and problematic smartphone use in college students

**DOI:** 10.3389/fpsyt.2025.1508756

**Published:** 2025-01-23

**Authors:** Wanying Xing, Xianyang Wang, Tingwei Feng, Jiaxin Xie, Chang Liu, Xiuchao Wang, Hui Wang, Lei Ren, Xufeng Liu

**Affiliations:** ^1^ Department of Military Medical Psychology, Air Force Medical University, Xi’ an, China; ^2^ BrainPark, School of Psychological Sciences, Turner Institute for Brain and Mental Health, Monash University, Clayton, VIC, Australia; ^3^ Military Psychology Section, Logistics University of People’s Armed Police Force, Tianjin, China; ^4^ Military Mental Health Services & Research Center, Tianjin, China

**Keywords:** social anxiety, problematic smartphone use, network analysis, sex difference, college students

## Abstract

**Background:**

Social anxiety (SA) and problematic smartphone use (PSU) have become increasingly common among college students in recent decades, with research indicating a mutual increase in risk. This study aim to deepen the understanding of how SA and PSU are interconnected at the symptom-level within this demographic using network analysis.

**Methods:**

We recruited 1,197 college students from four institutions in Shaanxi Province, China. Symptoms of SA and PSU were assessed through self-report questionnaires. A regularized Gaussian graphical model was used to estimate the relationships between these symptoms. We calculated Bridge Expected Influence (BEI) to identify key symptoms contributing to their co-occurrence. Additionally, a network comparison test was conducted to examine potential gender differences in the BEI values of the SA-PSU network.

**Results:**

Distinct relationships were observed between SA and PSU symptoms. Notably, the connections between ‘Get embarrassed very easily’ (SA3) and ‘shyness in new situations’ (PSU1), as well as between SA3 and ‘Escape or relieve negative moods’ (PSU8), showed the strongest inter-construct connections. SA3 and PSU8 were identified as the key symptoms contributing to the co-occurrence, with the highest BEI. Network comparison tests between males and females revealed no significant differences in global expected influence, between-community edges weights, and BEI.

**Conclusion:**

The key bridging symptoms this study identified supports the existing theories about the co-occurrence of SA and PSU, and contributes to understanding the underlying mechanisms. Our findings suggest that interventions targeting negative emotions in daily interactions could be effective in reducing PSU.

## Introduction

Social anxiety (SA) is defined as a chronic emotional disorder characterized by an irrational fear or anxiety in social situations where there is potential for scrutiny or negative evaluation by others (1). Its prevalence in Chinese college students is about 12-14%, and topped out at 33% worldwide according to the present studies. Problematic smartphone use (PSU) is defined as excessive use of smartphones phones with marked functional impairment and distress (2, 3). SA and PSU have both become increasingly prevalent and among college students, with a notable rise in recent studies, and they may have serious negative impacts on individual mental health, social functioning and academic development (4–9). Meanwhile, the concrete symptoms and subtypes of SA and PSU have been classified more specifically as the increased attention and exploration (10, 11). The concrete manifestation of SA involve a set of cognitive, emotional and behavioral characteristics (12). For instance, the performance subtype SA has been widely regard as a distinct subtype whose physiological responses to performance situations were extremely strong. Individuals with performance subtype of SA primarily manifest anxious when speaking to group and troubled when being watched, while individuals with other forms of SA may manifest shyness in new situations, nervous because large groups and hard to talk to strangers (13).

In recent years, there has been increasing empirical studies pointed out the positive correlation between SA and PSU (14–18). SA has been treated as the antecedent of PSU, which might cause a range of other negative consequences, such as emotional symptoms (e.g., depression and anxiety) and addiction-like symptoms (e.g., preoccupation, withdrawal and tolerance) (19–21). PSU was associated with similar behavioral and emotional problems, and could further worsen the SA symptoms in turn (20, 22–24).

Meanwhile, theoretical studies proposed several potential theoretical models to explain the interaction between SA and PSU (25, 26). These models are based on the premise that PSU serves as a compensatory mechanism for coping with underlying psychopathology instead of pathological in and of itself inherently (27, 28). For instance, based on the Compensatory Internet Use Theory (CIUT, 28), the excessive electronics use is probably meant to alleviate their negative emotion driven by stressful job and life. In addition, the excessive reassurance seeking has been regarded as the first pathway of integrative Pathways Model, which treated PSU as a maladaptive coping strategy for negative emotions and events (27, 29). Under this theory, people with high level of SA might treat PSU as a way to distract themselves from negative emotions caused by social interaction. In other words, the PSU is driven by negative reinforcement (30, 31). Moreover, the Pathways Model proposed other two pathways leading to the manifestation of PSU, impulsiveness and extraversion, corresponding to individuals with poor impulse control and a constant desire for communication and external stimulation, respectively (29, 30). This impulsiveness pathway is promoted by low self-control or impulse control, which results in increased addictive-like PSU symptoms, such as preoccupation, tolerance and unsuccessful control (30). Impulsivity is a traditional and predictive dimension of most of behavioral addictions (e.g., PSU, 32). It is suggested that the extraversion pathway is linked to a wide range of risky smartphone-related behaviors, which is corresponding to the consequence of reward and excitement-driven smartphone usage patterns, but some studies failed to found the significant positive relationship between extraversion and PSU (30, 33). Several studies which centered around PSU and SA as well as other anxiety disorders have also provided empirical support for these theories (24–26). However, these studies primarily explain the inter-connections leading to PSU through a single pathway, rather than exploring the multifaceted nature of these relationships from a more integrated direction.

Although numerous studies have provided empirical evidence on the association between SA and PSU, most of studies relied on aggregate scores (14–17, 34, 35). Both PSU and SA are heterogeneous syndromes, composed of distinct symptoms (1, 35–37). The aggregate scoring approach may conceal the unique contributions of individual SA and PSU symptoms, thereby limiting mechanistic insights into their co-occurrence. Shifting focus from the disorder level to the symptom level can yield more nuanced pathological information about the co-occurrence between PSU and SA (38–43).

Network analysis offers a symptom-level-oriented approach and achieves significant methodological benefits over traditional statistical models, such as intuitive visualization and unique indices (44, 45). It effectively visualizes the network structure between symptom communities with nodes and edges. As a symptom-level analysis, it also examines the direct relationships between each PSU symptom and specific SA symptoms, providing deeper theoretical and clinical insights. Furthermore, the bridge expected influence (BEI) index could identify key bridge symptoms that activate the other community in general and lead to the co-occurrence of SA and PSU (39). This is crucial for identifying potential intervention targets for managing their co-occurrence (41). It is worth mentioning that the lifetime prevalence of SA and clinical severity of SA is significantly higher among females than males (46, 47), while males are more prone to PSU than females (48). Male and female college students exhibit distinct preferences and usage patterns when it comes to smartphone functionality, with males more inclined towards entertainment uses (e.g., gaming and watching anime), while females more inclined towards social applications of smartphones (49). Therefore, a deeper inspection of the gender differences of the inter-connections between SA and PSU may help in targeted alleviation of its comorbidity, and the network comparison tests could be an appropriate method on this purpose.

Recent studies by Zhou and Shen (18) and Tao et al. (24) have examined the correlation and cross-lagged effects between SA and PSU symptoms among high school students using network analysis. Both studies identified ‘withdrawal’ as a bridge symptom linking SA and PSU. Tao et al. (24) also noted the potential impact of ‘prolonged online learning’ and the moderating role of ‘the fear of missing out’ in this process. However, the risk period for the onset of SA can extend to the early 40s (21). The significant impairments associated with SA and PSU in college students highlighted the need to understand the mechanisms in specific age groups (23, 50, 51). Compared to high school students, college students undergo substantial changes in their social networks and face increased social evaluation as they transition into social life (52, 53). Additionally, upon entering college, young adults often have unrestricted access to mobile phones, more free time, and less parental supervision. This freedom might make the virtual world a more secure environment for those who are more susceptible to anxiety and embarrassment in social interactions (20). Extending such research to include college students is thus crucial for developing better understanding and strategies to improve their mental health.

To address the previously mentioned issues, this study employed network analysis to explore the unique connections between SA and PSU symptoms in college students. Our aims are: 1) to identify the inter-connections between SA and PSU symptoms in this demographic; 2) to identify bridge nodes in this co-occurrence; and 3) to explore potential sex differences in the network characteristics.

## Methods

### Participants

The study recruited 1,432 college students via Wenjuanxing, an online tool, and used the convenience sampling approach. Invitations were distributed through WeChat. After excluding 235 responses due to incomplete demographic information (n = 51) and failure to pass two mandatory attention checks (n = 184), a final sample of 1,197 participants was determined. This group comprised 860 females, aged 18 to 22, with a mean age of 18.68 years (SD = 0.85). All participants provided informed consent at the beginning of the survey. The data collection and analysis procedures were approved by the Ethics Committee of the First Affiliated Hospital of the Air Force Medical University (Approval No. KY20234188-1, dated 21 May 2022) and adhered to the Declaration of Helsinki.

### Measures

#### SA symptoms

SA symptoms were assessed using the SA subscale of the Self-Consciousness Scale (SASS-CS; 54). This subscale includes six items, one of which is reverse coded for scoring. Participants rated each item on a 5-point scale from 1 (strongly disagree) to 5 (totally agree), where higher scores indicate more severe SA symptoms. The Chinese version of the SASS-CS has demonstrated acceptable reliability and validity (51, 55), and confirmed acceptable internal consistency (α = 0.85) in the current study.

#### PSU symptoms

PSU symptoms were assessed using the Problematic Smartphone Use Scale (PSUS) developed by Hussain et al. (56). This modified version includes nine items that comprehensively cover the addictive types of PSU symptoms (e.g. preoccupation, withdrawal, tolerance, and escapism/avoidance). Participants rated each item on a 5-point scale, from 1 (never) to 5 (very often). Consistent with previous studies (36, 57), the scale demonstrated good internal consistency (α = 0.85) in the current study.

### Statistical analysis

The current structure of the SA-PSU network was estimated using a combination of the Extended Bayesian Information Criterion Graphical Least Absolute Shrinkage and Selection Operator (EBICglasso) and the Gaussian Graphical Model (GGM), with a gamma value set at 0.5 (58). In this network, edges signify the partial (Spearman) correlations between node pairs, adjusted for other nodes influence (59). The network was constructed and visualized using the Fruchterman-Reingold algorithm, via the R-package qgraph (60, 61). The bridge expected influence (BEI) was calculated via the R-package networktools to evaluate each node’s bridging effect. Higher positive BEI values suggest stronger ability to activate another community, whereas higher negative values indicate a greater ability to inactivate another community (41).

To evaluate the accuracy of edge weights, we calculated 95% confidence intervals (CIs) of edge weights using 1,000 bootstrap samples, and conducted bootstrapped difference tests. We assessed the stability of the BEI estimation by calculating the Correlation Stability (CS) coefficient through a case-dropping methodology, using 1,000 bootstrap samples as well. According to Epskamp et al. (58), a CS coefficient above 0.5 is considered optimal. We conducted these analyses using the R-package bootnet.

To explore potential sex differences within the network, we conducted the network comparison test using the R-package Network Comparison Test, with 1,000 permutations (62). We focused on gender differences in three key network characteristics: global expected influence (the sum of all edges in male and female networks), weights of between-community edges and node BEI. We applied the Bonferroni-Holm method to adjust the significance levels in order to account for multiple comparisons and thereby correct for family-wise errors. (van Borkulo et al., 2023). Moreover, due to the disparity in the number of male and female participants, a subsample matching the number of male participants was randomly drawn from all female participants to undergo the same network comparison procedures, thereby validating the stability of the current network comparison results.

## Results

**Figure 1:**
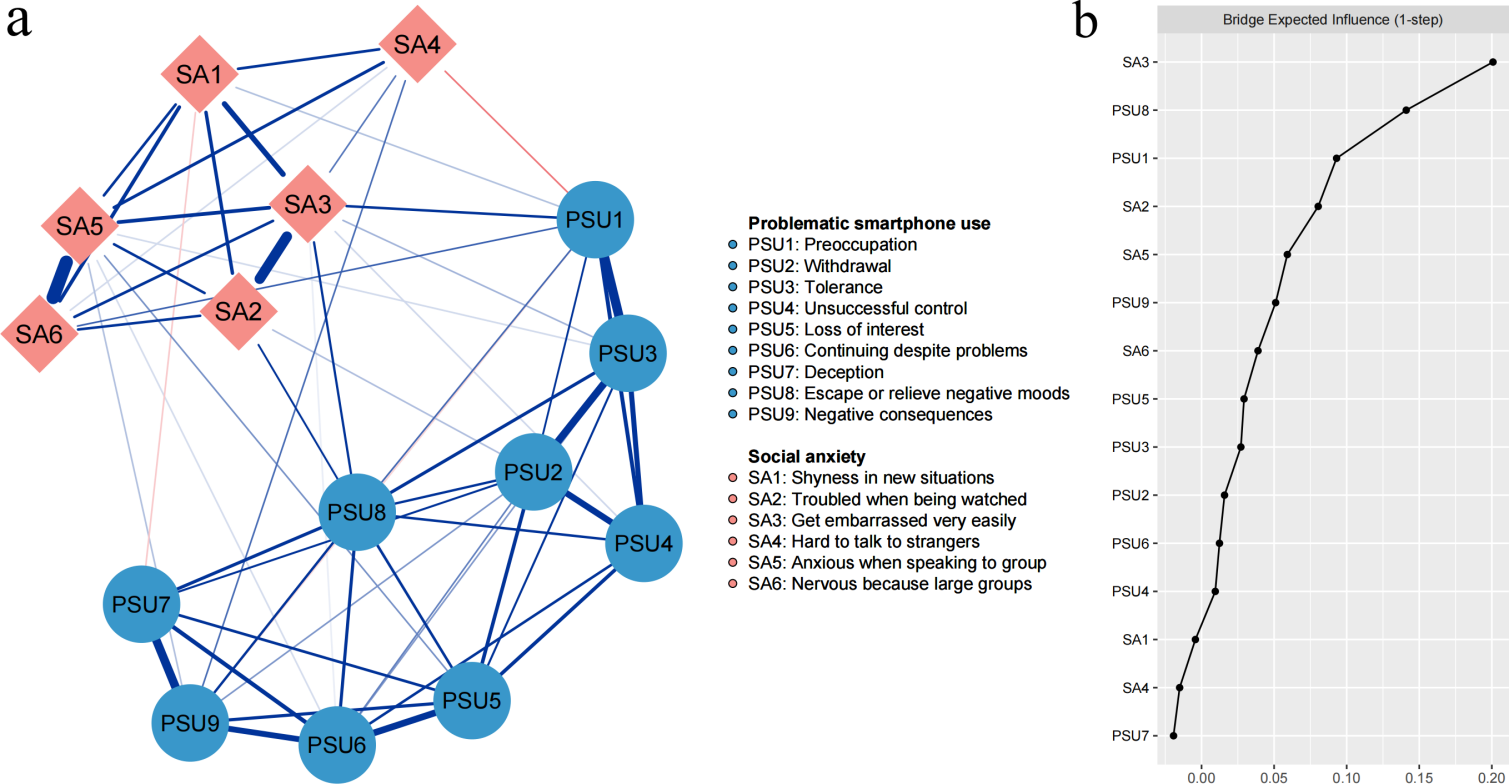
**(A)** The network structure of social anxiety and problematic smartphone use. Blue edges represent positive connections and red edges represent negative connections. **(B)** Bridge expected influence plot.

**Figure 1A** represents the final network structure of SA and PSU symptoms. A total of 17 between-community edges (31.48%) were generated out of 54 potential between-community edges, with edge weights ranging from -0.05 to 0.09. **Supplementary Table S1** (in the **Supplementary Materials**) shows all edge weights within the final network. ‘Troubled when being watched’ (SA2) is positively linked with three PSU symptoms and the strongest positive edge was between SA2 and ‘Escape or relieve negative moods’ (PSU8) (weight = 0.06). ‘Get embarrassed very easily’ (SA3) is positively linked with five PSU symptoms, among which two strongest positive edges were between SA3 and ‘Preoccupation’ (PSU1) (weight = 0.09) and symptom PSU8 (weight = 0.08). ‘Hard to talk to strangers’ (SA4) is negatively linked with symptom PSU1 (weight = -0.05) and positively linked with ‘Negative consequences’ (PSU9) (weight = 0.04). The bootstrapped 95% confidence intervals for the edge weights are shown in **Supplementary Figure S1**, while the bootstrapped difference test for edge weights is displayed in **Supplementary Figure S2** (both in the **Supplementary Materials 1**).

**Table 1** and **Figure 1B** present each node’s BEI. The nodes SA3 and PSU8 exhibited the highest positive BEI values, at 0.20 and 0.14, respectively. Conversely, ‘Deception’ and SA4 displayed negative BEI, both at -0.02. The CS-coefficient of BEI was 0.67, surpassing the threshold of 0.50 (**Supplementary Figure S3** in the **Supplementary Material 1**). Additionally, the bootstrapped difference test, detailed in **Supplementary Figure S4** (in the **Supplementary Material 1**), highlighted significant variations in BEI among the nodes. Notably, the BEI of SA3 was greater than those of all other nodes, while PSU8 significantly differed from all other nodes within the PSU community, except for PSU1.

**Table 1:** Abbreviations, mean scores, standard deviations and raw value of bridge expected influences for each variable selected in the present network.

Variables	Abbr	M	SD	BEI
SA symptoms				
Shyness in new situations	SA1	3.08	1.21	-0.004
Troubled when being watched	SA2	3.03	1.17	0.08
Get embarrassed very easily	SA3	2.92	1.20	0.20
Hard to talk to strangers	SA4	2.62	1.16	-0.02
Anxious when speaking to group	SA5	3.01	1.17	0.06
Nervous because large groups	SA6	3.28	1.20	0.04
PSU symptoms				
Preoccupation	PSU1	2.91	1.17	0.09
Withdrawal	PSU2	2.02	1.10	0.02
Tolerance	PSU3	2.39	1.17	0.03
Unsuccessful control	PSU4	2.22	1.16	0.01
Loss of interest	PSU5	1.86	0.99	0.03
Continuing despite problems	PSU6	1.76	0.96	0.01
Deception	PSU7	1.47	0.79	-0.02
Escape or relieve negative moods	PSU8	2.17	1.17	0.14
Negative consequences	PSU9	1.50	0.80	0.05

Abbr, Abbreviation; M, mean; SD, standard deviation; BEI, bridge expected influence.

**Figure 2:**
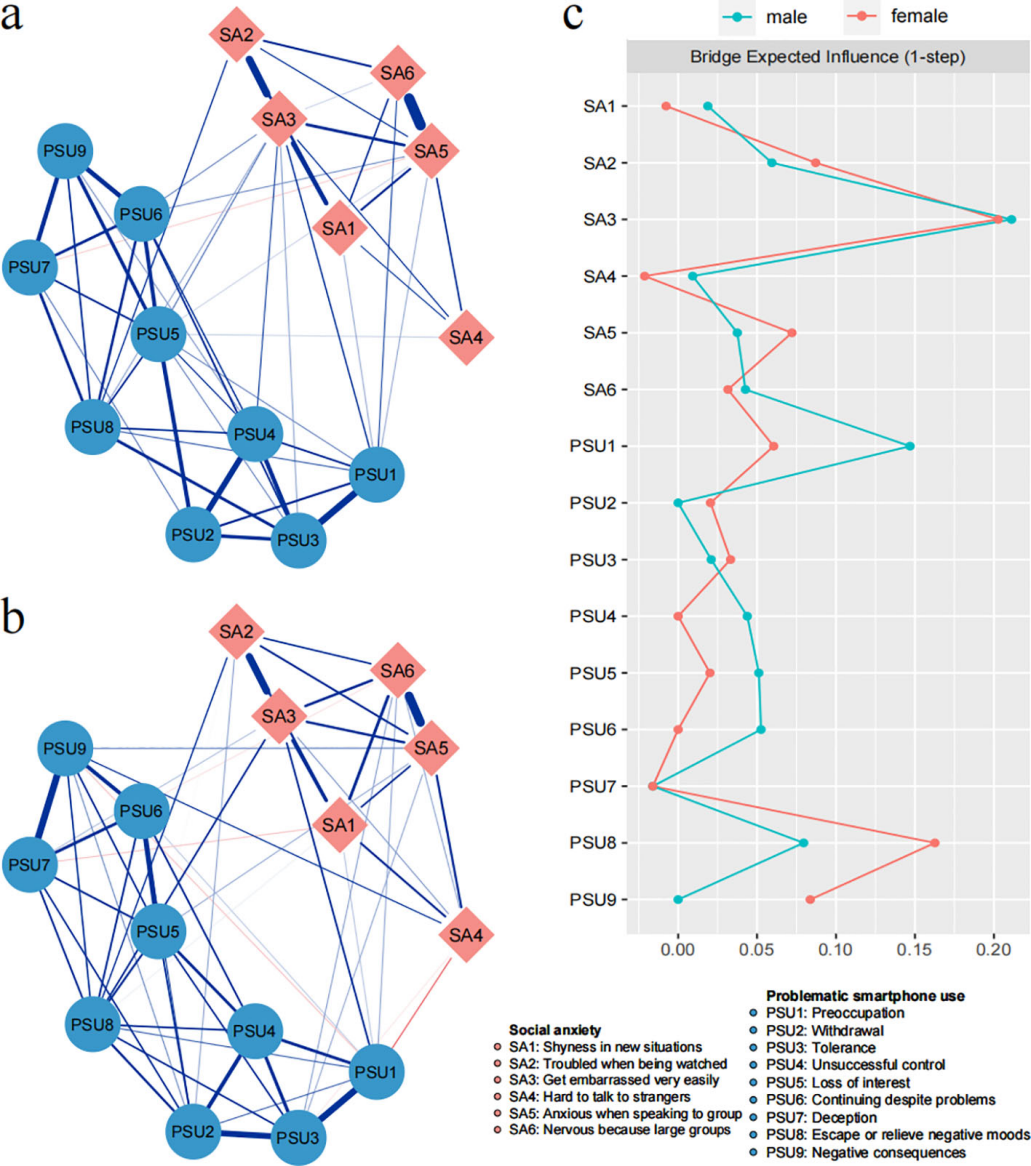
Network structure of social anxiety symptoms and problematic smartphone use symptoms for male **(A)** and female **(B)** participants. Blue edges represent positive connections and red edges represent negative connections. **(C)** Bridge expected influence plots for male and female participants.

**Figure 2A** shows the network structure for male participants, and **Figure 2B** shows the corresponding structure for females. Network comparison tests between males and females revealed no significant differences in global expected influence ([S] = 0.10; males = 6.51, females = 6.61; *p* = 0.30), network invariance ([M] = 0.15; *p* = 0.46), and BEI. **Figure 2C** shows the BEI values for female and male networks. **Supplementary Figure S5** and **Supplementary Figure S6** (in the **Supplementary Material 1**) show the robustness test results of female and male network. The CS-coefficients of edges in both female and male networks are 0.75, indicating the stabilities of edge weights are adequate. The CS-coefficients of BEI are 0.59 in female network and 0.44 in male network. Additionally, the network comparison tests on the extracted smaller subsample also showed the same results, indicating the results based on original data set was stable (in the **Supplementary Material 2**).

## Discussion

This study investigated the connections between SA and PSU symptoms in college students using network analysis. Our findings revealed distinct connections between SA and PSU symptoms, with the strongest links observed between SA3 and PSU1; as well as between SA3 and PSU8. Additionally, SA3 demonstrated the strongest positive bridging effect on the PSU symptoms community, while symptom PSU8 showed the strongest positive bridging effect on the SA symptoms community. Gender had minimal impact on the network characteristics.

We observed that SA2 and SA3 were positively related to the same PSU symptom PSU8. It supported previous theories about the mechanisms underlying PSU. This correlation can be explained by negative reinforcement and the excessive reassurance pathway of the Pathways Model: college students with high SA may use smartphones as a coping strategy to mitigate negative emotions from interpersonal interactions (29, 63). Recent studies suggested that frequent cell phone use might be a habitual coping style for avoiding anxiety, and that individuals with SA tended to exhibit more rigid regulation patterns (18, 64). The fear and anxiety about missing out on rewarding events may directly trigger the increase of smartphone use frequency (65, 66). Our findings aligned with these prior research results and underscored the importance of adopting more appropriate and flexible emotional regulation strategies rather than the avoidance coping strategies like PSU in individuals with high levels of SA.

In addition, SA3 positively linked with the PSU1, which could be explained by the excessive reassurance pathway and the impulsiveness pathway of the Pathway Model (27, 29). The more often individuals feel embarrassed in a social situation, the more likely they are to be preoccupied with smartphone use. The imbalance between the effort they put in (i.e., keep calm and restrain negative emotions) and the unsatisfactory outcome they received frequently in social interaction would cause negative emotional overreaction (i.e., embarrassment, anxiety, frustration and other negative emotions), which leads to a decreased performance of self-control (i.e., engage in preoccupation with smartphone use) (67, 68). Similarly, the positive edges between SA symptoms (i.e., characterized by being nervous in large groups, shyness in new situations) and PSU1 supported the same pathway. Previous studies have also provided numerous evidences of the positive symptom-level associations between SA and PSU (17, 18), while the current study further supported that those college students with high level of SA could be more likely to achieve their aim of social avoidance and relieve irritability (e.g., embarrassment, tension and shyness) through excessive focus on their smartphones. It might be helpful to improve the self-control of SA individuals in smartphone usage by realizing the real motivation of their behavior. Furthermore, by revealing a nuanced connection that specific SA symptoms linked to PSU symptoms (i.e., Preoccupation) differently, we highlighted the specific symptom that might drive the SA-PSU association. The current findings built upon this knowledge revealed a nuanced connection: different SA subtype (i.e., performance subtype of SA) may be more closely linked to specific PSU symptoms.

Intriguingly, we found that SA4 was negatively related to PSU1 but positively associated with PSU9. One possible explanation for this could be: difficulty in talking to strangers may reduce smartphone preoccupation as individuals who are less comfortable engaging with new people might not use social media extensively to build and expand their networks, resulting in less overall screen time. However, this same discomfort can lead to negative consequences. The reluctance to initiate conversations, both online and in-person, can result in negative consequences (e.g., missed opportunities and limited personal and professional growth) due to limited social and professional connectivity.

The node bridge centrality of the SA-PSU network may cast light on the prominent role of specific SA symptoms in the development and maintenance of PSU. Since that BEI of SA3 is significantly greater than other nodes in the SA symptoms community, it was identified as bridge nodes for the SA-PSU association. It suggested that SA3 had significantly stronger associations with the majority PSU symptoms than other SA symptoms. Furthermore, in the PSU symptoms community, PSU8 was identified as the bridge node, which indicated that PSU8 might be susceptible to the SA symptoms community. The bridge nodes for the SA-PSU association reported in the current study were different from several studies reported recently (i.e., withdrawal or productivity loss), which might due to the age difference of participants (24, 35). These results might emphasize the need of targeted interventions for different age groups. For instance, the acceptance and commitment therapy or stand-alone virtual reality exposure therapy (69, 70) might be more valid for reducing co-occurrence of SA and PSU among college students, as the coping-motivated smartphone use are more likely in college students as previously stated.

Although prior studies have suggested that SA and PSU may be affected by sex independently (46, 71, 72), the sex differences have seldom been focused or reported in the studies of their comorbidity (20, 34, 35, 73). Therefore, the sex differences have been explored in the current study specifically, and our results indicated no significant sex differences on the SA-PSU connection, or the bridge centrality in college students. Thus, the comparison of network between sexes underscored more caution in attributing sex differences to these connections, given the current scarcity of robust evidence. Future investigations should aim to consolidate these findings by gathering additional evidence, thereby enabling the formulation of more robust and convincing conclusions. This will not only enrich our theoretical knowledge but also pave the way for more targeted and effective clinical interventions across diverse populations.

The study emphasized the importance of targeted interventions based on the complex connections between PSU and SA among college students. It was suggested that the co-occurrence mechanisms of PSU and SA among college students, as distinct from its among adolescent, necessitated tailored interventions that specifically target different ages (18, 24). Specifically, college students who have the most difficulty in regulating the negative emotions in interpersonal interactions exhibit a greater tendency of PSU problems. Therefore, a more appropriate way to regulate emotions is crucial coping strategies. Furthermore, this study highlighted the significance of SA3 as a significant predictor of symptom communities, underscoring the cultivation of self-regulation flexibility and self-consciousness in colleges and universities (74). To address SA and manage PSU more effectively, and to promote the physical and mental health of college students, interventions for the improvement of emotion regulation ability and adaptive strategies are warranted.

The current study has several limitations that warrant further consideration. Firstly, the findings were based on data from college students, potentially limiting their clinical applicability. Future research is required to examine these findings in clinical samples. Secondly, the cross-sectional design of this study limited our ability to establish the direction of associations between SA and PSU. The discussion, which considered SA as a risk factor for PSU, was primarily based on theoretical grounds (27, 28, 30). Longitudinal studies are necessary to determine the directionality of the observed relationships. Finally, our results may suggested that the interaction between PSU and SA might vary depending on the purpose of smartphone use. It is essential to distinguish between social and non-social PSU when exploring comorbidity issues.

## Conclusion

In the current study, we used a symptom-level network approach to explore the relationships between SA and PSU among college students. Our findings not only supported existing theoretical models (e.g. the Pathways Model) but also provided novel insights (e.g. negative reinforcement, smartphone use for social purpose) into the mechanisms behind the co-occurrence of these two constructs. Notably, the bridge centrality results emphasized the significant roles of SA3 and PSU8 in linking SA and PSU among college students. These findings could guide tailored campus prevention and intervention for SA and PSU among this specific age group. For instance, teaching students to cope flexibly with negative moods in social contexts could effectively prevent the worsening of PSU.

## Data Availability

The raw data supporting the conclusions of this article will be made available by the authors, without undue reservation.
