# Supplementary material for: Network analysis of the association between social anxiety and problematic smartphone use in college students
Source: Front Psychiatry. 2025 Jan 23;16:1508756. doi: 10.3389/fpsyt.2025.1508756 (PMC11799245; doi:10.3389/fpsyt.2025.1508756)
Supplement: Supplementary file 1 [file DataSheet1.pdf]

Supplementary Materials

1. Table S1. All edges weights within the final network
2. Figure S1. Accuracy of edge weights
3. Figure S2. Bootstrapped difference test for edge weights
4. Figure S3. Stability of the bridge expected influence
5. Figure S4. Bootstrapped difference test for the bridge expected influence
6. Figure S5. Robustness test of female SA-PSU network.
7. Figure S6. Robustness test of male SA-PSU network

Table S1. All edges weights within the final network

	SA1	SA2	SA3	SA4	SA5	SA6	PSU1	PSU2	PSU3	PSU4	PSU5	PSU6	PSU7	PSU8	PSU9
**SA1**	0.000	0.131	0.219	0.098	0.097	0.140	0.015	0.000	0.000	0.000	0.000	0.000	-0.019	0.000	0.000
**SA2**	0.131	0.000	0.444	0.000	0.092	0.092	0.000	0.016	0.000	0.000	0.002	0.000	0.000	0.062	0.000
**SA3**	0.219	0.444	0.000	0.033	0.143	0.099	0.090	0.000	0.018	0.009	0.000	0.004	0.000	0.079	0.000
**SA4**	0.098	0.000	0.033	0.000	0.115	0.009	-0.051	0.000	0.000	0.000	0.000	0.000	0.000	0.000	0.036
**SA5**	0.097	0.092	0.143	0.115	0.000	0.572	0.000	0.000	0.009	0.000	0.027	0.008	0.000	0.000	0.015
**SA6**	0.140	0.092	0.099	0.009	0.572	0.000	0.039	0.000	0.000	0.000	0.000	0.000	0.000	0.000	0.000
**PSU1**	0.015	0.000	0.090	-0.051	0.000	0.039	0.000	0.061	0.395	0.153	0.000	0.000	0.000	0.037	-0.015
**PSU2**	0.000	0.016	0.000	0.000	0.000	0.000	0.061	0.000	0.308	0.260	0.138	0.031	0.069	0.083	0.025
**PSU3**	0.000	0.000	0.018	0.000	0.009	0.000	0.395	0.308	0.000	0.203	0.070	0.025	0.000	0.120	0.000
**PSU4**	0.000	0.000	0.009	0.000	0.000	0.000	0.153	0.260	0.203	0.000	0.137	0.093	0.000	0.088	0.000
**PSU5**	0.000	0.002	0.000	0.000	0.027	0.000	0.000	0.138	0.070	0.137	0.000	0.276	0.099	0.095	0.119
**PSU6**	0.000	0.000	0.004	0.000	0.008	0.000	0.000	0.031	0.025	0.093	0.276	0.000	0.164	0.115	0.238
**PSU7**	-0.019	0.000	0.000	0.000	0.000	0.000	0.000	0.069	0.000	0.000	0.099	0.164	0.000	0.121	0.362
**PSU8**	0.000	0.062	0.079	0.000	0.000	0.000	0.037	0.083	0.120	0.088	0.095	0.115	0.121	0.000	0.084
**PSU9**	0.000	0.000	0.000	0.036	0.015	0.000	-0.015	0.025	0.000	0.000	0.119	0.238	0.362	0.084	0.000

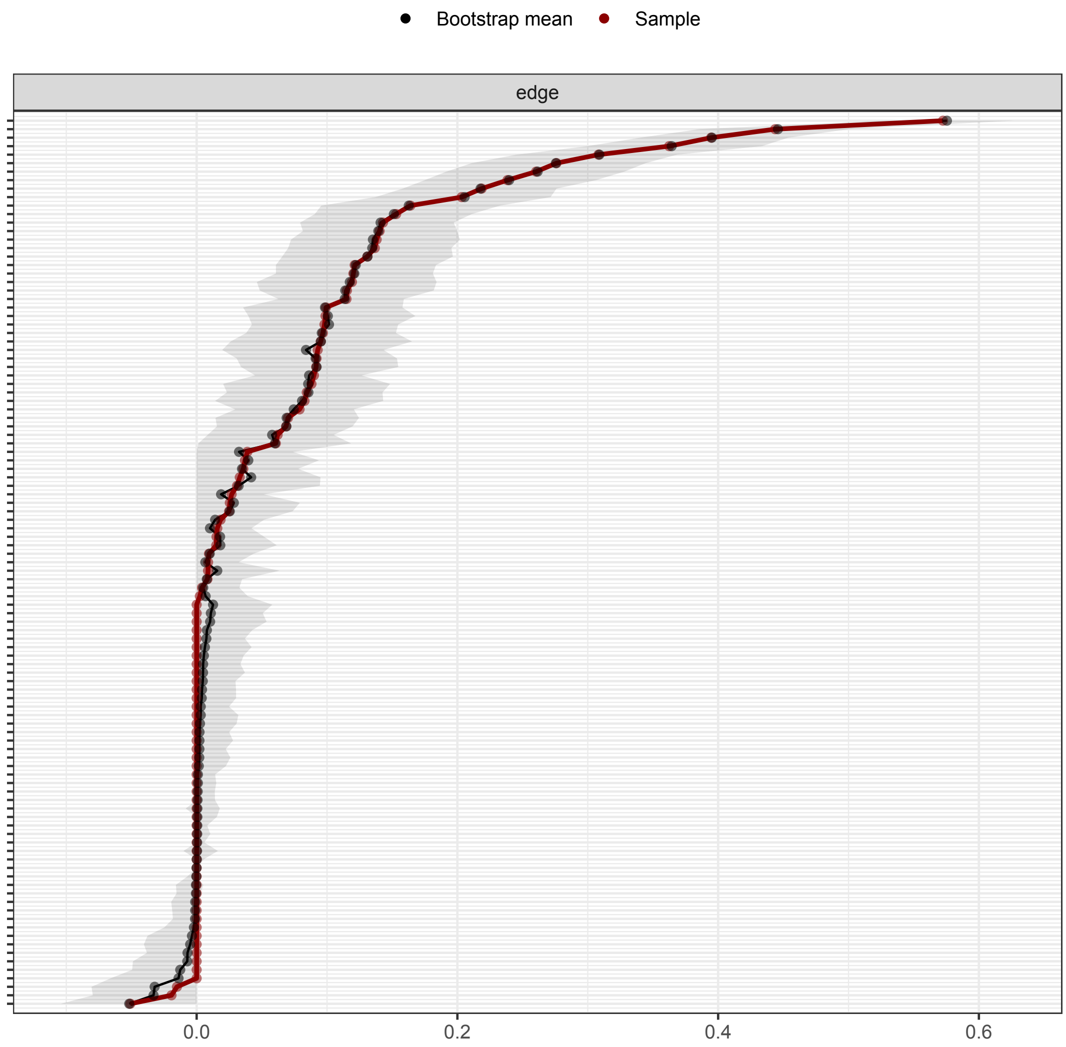

Figure S1. Accuracy of edge weights

Note: The red line depicts the sample edge weights and the gray bar depicts the bootstrapped confidence interval.

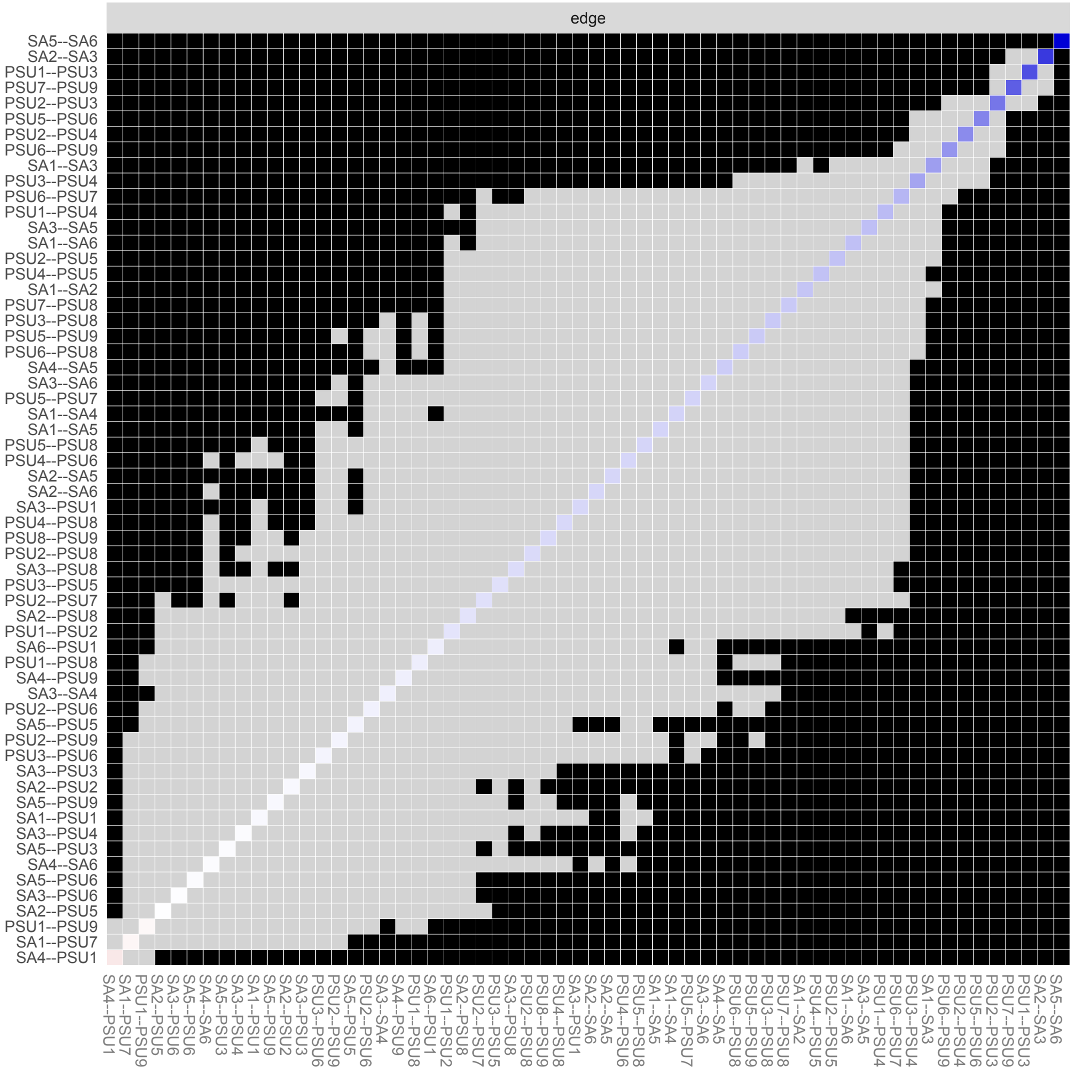

Figure S2. Bootstrapped difference test for edge weights

Note: Gray boxes indicate edge weights that do not differ significantly from one another, while black boxes indicate edge weights that do differ significantly. Blue and red boxes on the diagonal correspond to edge weights with positive and negative correlations, respectively.

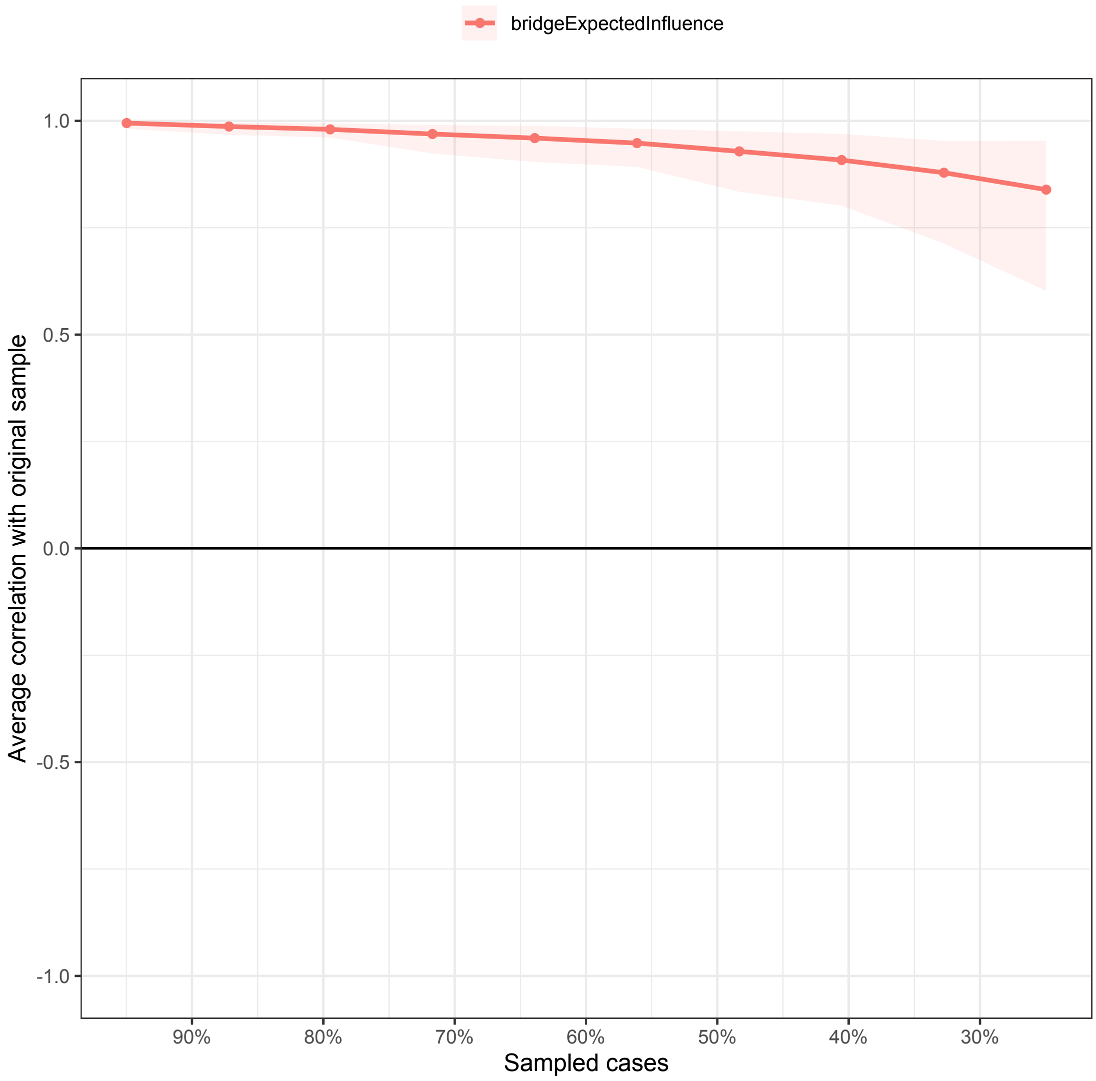

Figure S3. Stability of the bridge expected influence values

Note: The red bar represents the average correlation between bridge expected influence.

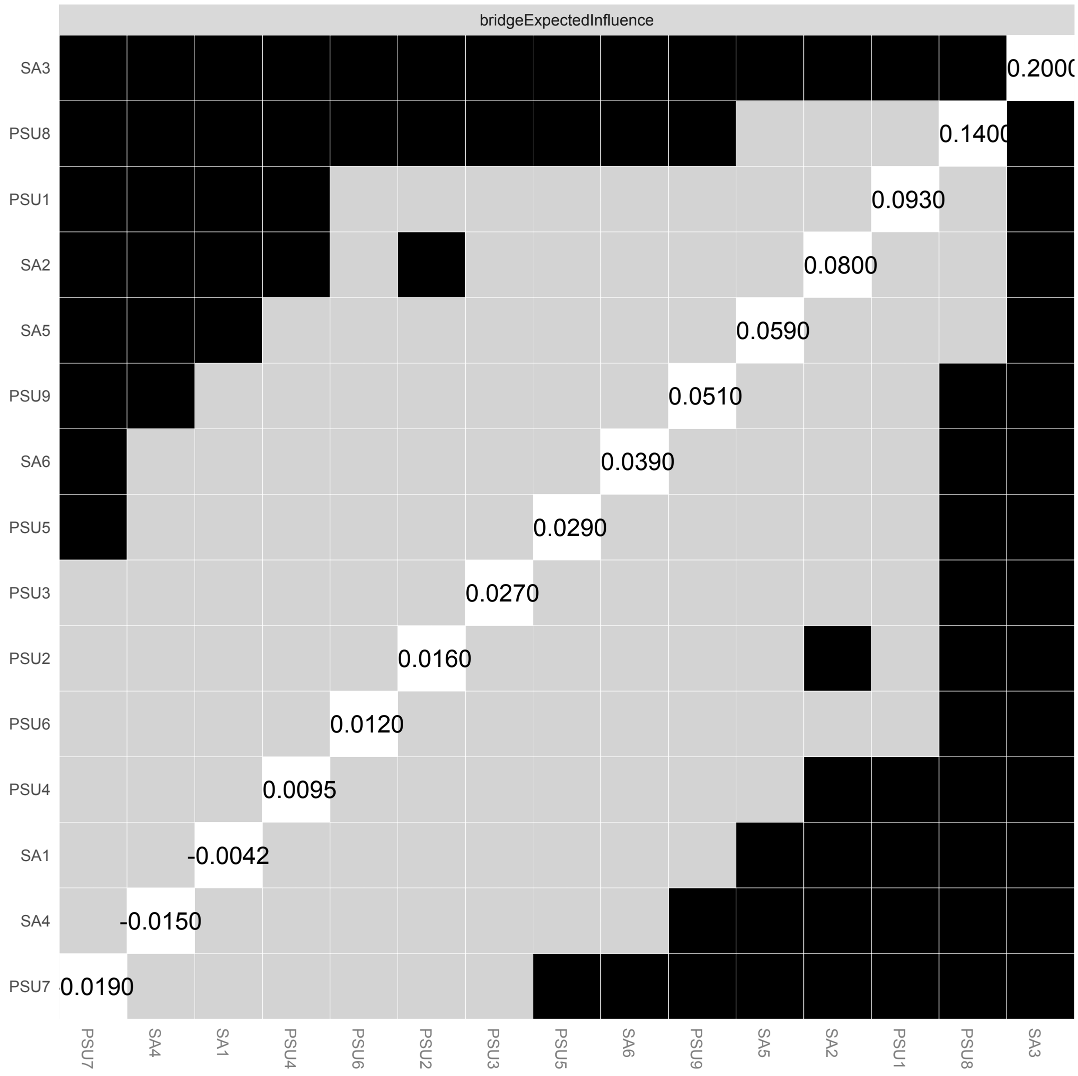

Figure S4. Bootstrapped difference test for the bridge expected influence values

Note: Gray boxes indicate the bridge expected influence that do not differ significantly from one another, while black boxes indicate the bridge expected influence that do differ significantly. The number in the white boxes (i.e., diagonal line) represent the value of the bridge expected influence.

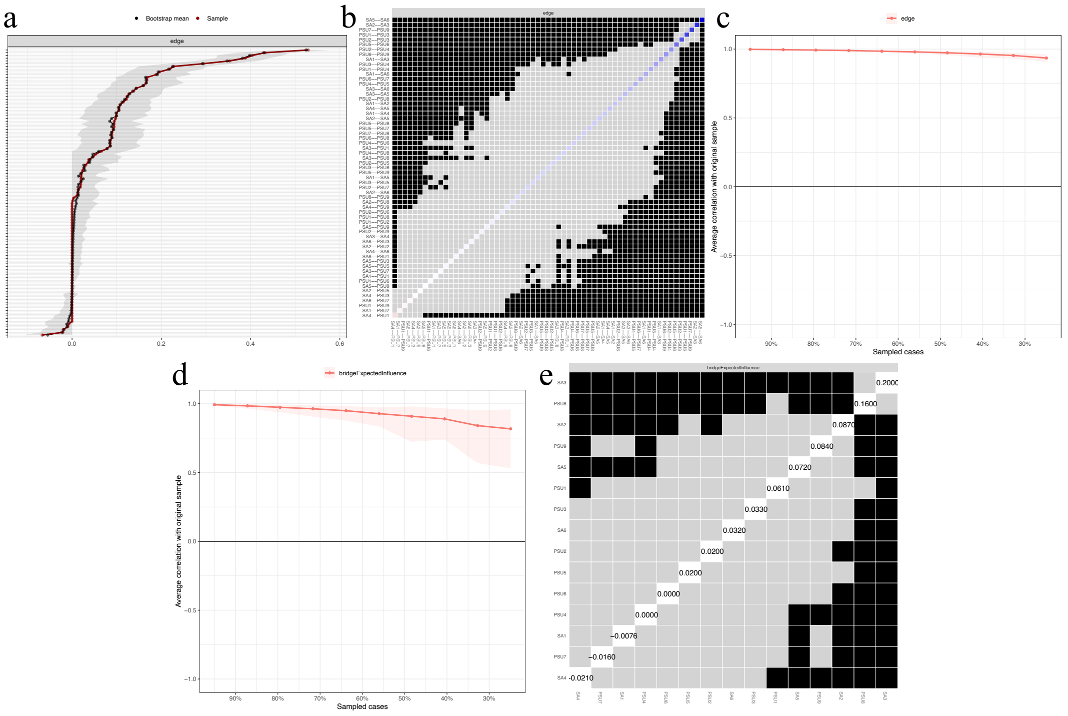

Figure S5. Robustness test of female SA-PSU network

Note: (a) Accuracy of edge weights. The red line depicts the sample edge weights and the gray bar depicts the bootstrapped confidence interval. (b) Bootstrapped difference test for edge weights. Gray boxes indicate edge weights that do not differ significantly from one another, while black boxes indicate edge weights that do differ significantly. (c) Stability of the edge weight. Correlation stability coefficient = 0.75. The red bar represents the average correlation between edge weight. (d) Stability of the bridge expected influence values. Correlation stability coefficient = 0.59. The red bar represents the average correlation between bridge expected influence. (e) Bootstrapped difference test for the bridge expected influence values. Gray boxes indicate the bridge expected influence that do not differ significantly from one another, while black boxes indicate the bridge expected influence that do differ significantly.

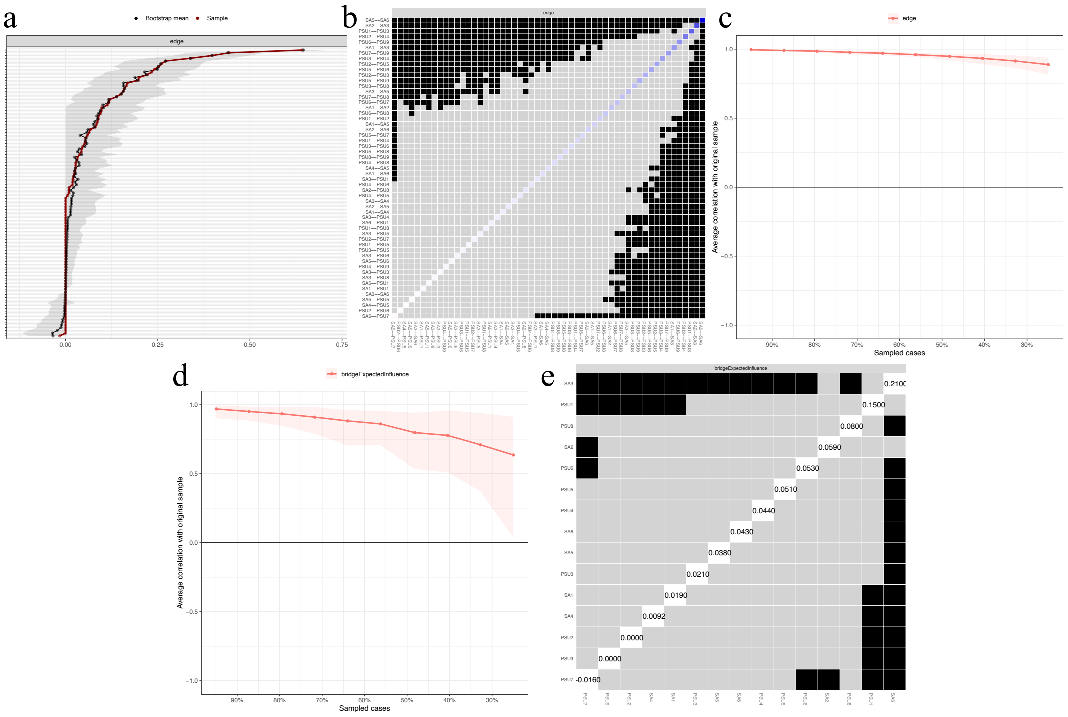

Figure S6. Robustness test of male SA-PSU network

Note: (a) Accuracy of edge weights. The red line depicts the sample edge weights and the gray bar depicts the bootstrapped confidence interval. (b) Bootstrapped difference test for edge weights. Gray boxes indicate edge weights that do not differ significantly from one another, while black boxes indicate edge weights that do differ significantly. (c) Stability of the edge weight. Correlation stability coefficient = 0.75. The red bar represents the average correlation between edge weight. (d) Stability of the bridge expected influence values. Correlation stability coefficient = 0.44. The red bar represents the average correlation between bridge expected influence. (e) Bootstrapped difference test for the bridge expected influence values. Gray boxes indicate the bridge expected influence that do not differ significantly from one another, while black boxes indicate the bridge expected influence that do differ significantly.
