# Supplementary material for: Network analysis of the association between social anxiety and problematic smartphone use in college students
Source: Front Psychiatry. 2025 Jan 23;16:1508756. doi: 10.3389/fpsyt.2025.1508756 (PMC11799245; doi:10.3389/fpsyt.2025.1508756)
Supplement: Supplementary file 2 [file DataSheet2.pdf]

Original data results （$N_{male} = 337$, $N_{female} = 860$）

NETWORK INVARIANCE TEST
Test statistic M:
0.1546782
p-value 0.4575425

GLOBAL EXPECTED INFLUENCE INVARIANCE TEST
Global EI per group: 6.51212 6.614028
Test statistic S: 0.1019085
p-value 0.2997003

EDGE INVARIANCE TEST

	Var1	Var2	p-value	Test statistic E
16	SA1	SA2	1.0000000	0.02705835
31	SA1	SA3	0.9877622	0.06420213
32	SA2	SA3	1.0000000	0.01083237
46	SA1	SA4	0.9508234	0.06791363
47	SA2	SA4	1.0000000	0.00000000
48	SA3	SA4	1.0000000	0.02979675
61	SA1	SA5	1.0000000	0.02835719
62	SA2	SA5	1.0000000	0.05568857
63	SA3	SA5	1.0000000	0.02673387
64	SA4	SA5	1.0000000	0.03780405
76	SA1	SA6	0.8223776	0.08726902
77	SA2	SA6	1.0000000	0.02746160
78	SA3	SA6	0.6818182	0.13390032
79	SA4	SA6	1.0000000	0.02033461
80	SA5	SA6	0.7237762	0.11244002
91	SA1	PSU1	1.0000000	0.00604224
92	SA2	PSU1	1.0000000	0.00000000
93	SA3	PSU1	1.0000000	0.02828254
94	SA4	PSU1	0.8223776	0.06682227
95	SA5	PSU1	0.8223776	0.01892080
96	SA6	PSU1	1.0000000	0.02283057
106	SA1	PSU2	1.0000000	0.00000000
107	SA2	PSU2	1.0000000	0.02049401
108	SA3	PSU2	1.0000000	0.00000000
109	SA4	PSU2	1.0000000	0.00000000
110	SA5	PSU2	1.0000000	0.00000000
111	SA6	PSU2	1.0000000	0.00000000
112	PSU1	PSU2	0.8223776	0.08321695
121	SA1	PSU3	1.0000000	0.00000000

122	SA2	PSU3	1.0000000	0.00000000
123	SA3	PSU3	1.0000000	0.02091538
124	SA4	PSU3	1.0000000	0.00743218
125	SA5	PSU3	0.8354146	0.01935222
126	SA6	PSU3	0.8223776	0.02130318
127	PSU1	PSU3	1.0000000	0.00975010
128	PSU2	PSU3	0.6818182	0.15467823
136	SA1	PSU4	1.0000000	0.00000000
137	SA2	PSU4	1.0000000	0.00000000
138	SA3	PSU4	0.7237762	0.04381295
139	SA4	PSU4	1.0000000	0.00000000
140	SA5	PSU4	1.0000000	0.00000000
141	SA6	PSU4	1.0000000	0.00000000
142	PSU1	PSU4	0.9821996	0.07103484
143	PSU2	PSU4	0.8223776	0.11014988
144	PSU3	PSU4	1.0000000	0.05269041
151	SA1	PSU5	1.0000000	0.00000000
152	SA2	PSU5	1.0000000	0.00134721
153	SA3	PSU5	0.7237762	0.03264060
154	SA4	PSU5	0.8223776	0.00917938
155	SA5	PSU5	1.0000000	0.00951043
156	SA6	PSU5	1.0000000	0.00000000
157	PSU1	PSU5	0.8223776	0.02759116
158	PSU2	PSU5	0.7237762	0.14138616
159	PSU3	PSU5	0.9821996	0.05861219
160	PSU4	PSU5	0.8223776	0.10630679
166	SA1	PSU6	1.0000000	0.00000000
167	SA2	PSU6	1.0000000	0.00000000
168	SA3	PSU6	0.8223776	0.02720596
169	SA4	PSU6	1.0000000	0.00000000
170	SA5	PSU6	0.8223776	0.02532895
171	SA6	PSU6	1.0000000	0.00000000
172	PSU1	PSU6	1.0000000	0.01151706
173	PSU2	PSU6	1.0000000	0.04258377
174	PSU3	PSU6	0.6818182	0.09505864
175	PSU4	PSU6	1.0000000	0.03385785
176	PSU5	PSU6	0.9877622	0.07266196
181	SA1	PSU7	1.0000000	0.02037764
182	SA2	PSU7	1.0000000	0.00000000
183	SA3	PSU7	0.8861828	0.01390040
184	SA4	PSU7	1.0000000	0.00000000
185	SA5	PSU7	0.6818182	0.01602536
186	SA6	PSU7	0.8354146	0.00956697
187	PSU1	PSU7	1.0000000	0.00000000

188	PSU2	PSU7	0.9877622	0.05449586
189	PSU3	PSU7	1.0000000	0.00000000
190	PSU4	PSU7	1.0000000	0.00000000
191	PSU5	PSU7	1.0000000	0.00737895
192	PSU6	PSU7	1.0000000	0.00946261
196	SA1	PSU8	1.0000000	0.00000000
197	SA2	PSU8	1.0000000	0.00586713
198	SA3	PSU8	0.8223776	0.07392796
199	SA4	PSU8	1.0000000	0.00000000
200	SA5	PSU8	1.0000000	0.00330161
201	SA6	PSU8	1.0000000	0.00000000
202	PSU1	PSU8	1.0000000	0.00040191
203	PSU2	PSU8	0.7237762	0.12739574
204	PSU3	PSU8	0.8354146	0.07789081
205	PSU4	PSU8	1.0000000	0.00735619
206	PSU5	PSU8	1.0000000	0.01592195
207	PSU6	PSU8	1.0000000	0.03891787
208	PSU7	PSU8	1.0000000	0.05939232
211	SA1	PSU9	1.0000000	0.00000000
212	SA2	PSU9	1.0000000	0.00000000
213	SA3	PSU9	1.0000000	0.00000000
214	SA4	PSU9	0.8223776	0.05304862
215	SA5	PSU9	0.8223776	0.03062668
216	SA6	PSU9	1.0000000	0.00000000
217	PSU1	PSU9	1.0000000	0.01995482
218	PSU2	PSU9	1.0000000	0.02653811
219	PSU3	PSU9	1.0000000	0.00000000
220	PSU4	PSU9	0.9508234	0.02332033
221	PSU5	PSU9	0.8223776	0.10896151
222	PSU6	PSU9	1.0000000	0.05216263
223	PSU7	PSU9	0.7237762	0.15122537
224	PSU8	PSU9	1.0000000	0.01021281

CENTRALITY INVARIANCE TEST p-value

	bridgeExpectedInfluence
SA1	0.8091908
SA2	0.8091908
SA3	0.9611817
SA4	0.8091908
SA5	0.8091908
SA6	0.9394452
PSU1	0.5574426
PSU2	0.8091908
PSU3	0.9394452

PSU4	0.5574426
PSU5	0.8091908
PSU6	0.5574426
PSU7	0.9860140
PSU8	0.5574426
PSU9	0.5574426

Results from a random sample data of female（N_{male} = 337, N_{female} = 337）

NETWORK INVARIANCE TEST

Test statistic M:

0.2004159

p-value 0.2237762

GLOBAL EXPECTED INFLUENCE INVARIANCE TEST

Global EI per group: 6.51212 6.584371

Test statistic S: 0.07225103

p-value 0.5064935

EDGE INVARIANCE TEST

	Var1	Var2	p-value	Test statistic E
16	SA1	SA2	1.0000000	0.00550028
31	SA1	SA3	0.9320679	0.08356781
32	SA2	SA3	1.0000000	0.03710694
46	SA1	SA4	1.0000000	0.06054306
47	SA2	SA4	1.0000000	0.00000000
48	SA3	SA4	1.0000000	0.01198238
61	SA1	SA5	0.9145542	0.09127728
62	SA2	SA5	0.8868404	0.10495150
63	SA3	SA5	1.0000000	0.00478364
64	SA4	SA5	1.0000000	0.01595484
76	SA1	SA6	0.8868404	0.12860513
77	SA2	SA6	0.9320679	0.08128030
78	SA3	SA6	0.3409091	0.18108576
79	SA4	SA6	1.0000000	0.00000000
80	SA5	SA6	0.8868404	0.10206449
91	SA1	PSU1	1.0000000	0.00835768
92	SA2	PSU1	1.0000000	0.00000000
93	SA3	PSU1	1.0000000	0.03149314
94	SA4	PSU1	0.8868404	0.09510854
95	SA5	PSU1	1.0000000	0.01892080
96	SA6	PSU1	0.9145542	0.04264900
106	SA1	PSU2	1.0000000	0.00000000
107	SA2	PSU2	1.0000000	0.00000000
108	SA3	PSU2	1.0000000	0.00000000
109	SA4	PSU2	1.0000000	0.00000000
110	SA5	PSU2	1.0000000	0.00000000
111	SA6	PSU2	1.0000000	0.00000000
112	PSU1	PSU2	0.8868404	0.09716531
121	SA1	PSU3	1.0000000	0.00000000

122	SA2	PSU3	1.0000000	0.00000000
123	SA3	PSU3	1.0000000	0.02091538
124	SA4	PSU3	0.9145542	0.03740789
125	SA5	PSU3	0.3409091	0.05836707
126	SA6	PSU3	1.0000000	0.00000000
127	PSU1	PSU3	1.0000000	0.01384222
128	PSU2	PSU3	0.3409091	0.19230345
136	SA1	PSU4	1.0000000	0.00000000
137	SA2	PSU4	1.0000000	0.00000000
138	SA3	PSU4	0.9145542	0.04381295
139	SA4	PSU4	1.0000000	0.00000000
140	SA5	PSU4	1.0000000	0.00000000
141	SA6	PSU4	1.0000000	0.00000000
142	PSU1	PSU4	1.0000000	0.06174948
143	PSU2	PSU4	0.8868404	0.13546912
144	PSU3	PSU4	1.0000000	0.02855761
151	SA1	PSU5	1.0000000	0.00000000
152	SA2	PSU5	1.0000000	0.00000000
153	SA3	PSU5	0.8391608	0.03264060
154	SA4	PSU5	1.0000000	0.00917938
155	SA5	PSU5	1.0000000	0.00929209
156	SA6	PSU5	1.0000000	0.00000000
157	PSU1	PSU5	1.0000000	0.02273971
158	PSU2	PSU5	0.8868404	0.10687873
159	PSU3	PSU5	0.9145542	0.07706266
160	PSU4	PSU5	1.0000000	0.02545637
166	SA1	PSU6	1.0000000	0.00000000
167	SA2	PSU6	1.0000000	0.00000000
168	SA3	PSU6	0.8868404	0.02720596
169	SA4	PSU6	0.9145542	0.04854238
170	SA5	PSU6	1.0000000	0.02532895
171	SA6	PSU6	1.0000000	0.00000000
172	PSU1	PSU6	0.9145542	0.00915463
173	PSU2	PSU6	1.0000000	0.02338632
174	PSU3	PSU6	0.3409091	0.09505864
175	PSU4	PSU6	1.0000000	0.02301030
176	PSU5	PSU6	0.8868404	0.11633308
181	SA1	PSU7	1.0000000	0.00000000
182	SA2	PSU7	1.0000000	0.00000000
183	SA3	PSU7	0.9320679	0.00223478
184	SA4	PSU7	1.0000000	0.00000000
185	SA5	PSU7	0.8868404	0.01602536
186	SA6	PSU7	1.0000000	0.00000000
187	PSU1	PSU7	1.0000000	0.00000000

188	PSU2	PSU7	1.0000000	0.04143312
189	PSU3	PSU7	1.0000000	0.00000000
190	PSU4	PSU7	1.0000000	0.00000000
191	PSU5	PSU7	1.0000000	0.05136356
192	PSU6	PSU7	1.0000000	0.05719560
196	SA1	PSU8	1.0000000	0.00000000
197	SA2	PSU8	1.0000000	0.01975851
198	SA3	PSU8	0.9145542	0.06844821
199	SA4	PSU8	1.0000000	0.00000000
200	SA5	PSU8	1.0000000	0.00000000
201	SA6	PSU8	1.0000000	0.00000000
202	PSU1	PSU8	1.0000000	0.03243879
203	PSU2	PSU8	0.9145542	0.07200857
204	PSU3	PSU8	0.8868404	0.09565013
205	PSU4	PSU8	0.8868404	0.10531239
206	PSU5	PSU8	1.0000000	0.03088540
207	PSU6	PSU8	1.0000000	0.05274723
208	PSU7	PSU8	1.0000000	0.05474587
211	SA1	PSU9	1.0000000	0.00000000
212	SA2	PSU9	1.0000000	0.00000000
213	SA3	PSU9	1.0000000	0.00000000
214	SA4	PSU9	0.9145542	0.02693834
215	SA5	PSU9	0.8868404	0.03019060
216	SA6	PSU9	1.0000000	0.00000000
217	PSU1	PSU9	1.0000000	0.00000000
218	PSU2	PSU9	1.0000000	0.00000000
219	PSU3	PSU9	0.8868404	0.01806132
220	PSU4	PSU9	0.8868404	0.02332033
221	PSU5	PSU9	0.8868404	0.14948710
222	PSU6	PSU9	1.0000000	0.06128466
223	PSU7	PSU9	0.4825175	0.20041595
224	PSU8	PSU9	1.0000000	0.03059163

CENTRALITY INVARIANCE TEST p-value

	bridgeExpectedInfluence
SA1	1.0000000
SA2	1.0000000
SA3	1.0000000
SA4	0.6256244
SA5	0.6256244
SA6	0.6256244
PSU1	0.6256244
PSU2	1.0000000
PSU3	1.0000000

PSU4	0.6256244
PSU5	0.6256244
PSU6	1.0000000
PSU7	0.8458208
PSU8	0.6256244
PSU9	0.6256244
